# Towards a minimum viable dataspace for the CEDS public sector: An initial implementation

**DOI:** 10.1016/j.dib.2026.112889

**Published:** 2026-05-28

**Authors:** Anastasios Nikolakopoulos, Georgia Dede, Charalampos Ipektsidis, Nikolaos Kefalakis, Petros Zervoudakis, Filodamos Papanatsios

**Affiliations:** a Research & Innovation Development Department, Netcompany SEE & EUI, Luxembourg, Luxembourg; b Department of Informatics & Telematics, Harokopio University, Athens, 17676, Greece

**Keywords:** Minimum Viable Dataspace, Common European Data Spaces, CEDS, Data Spaces, Public Administration, Secure Data Exchange, Data Sovereignty

## Abstract

Data sharing across borders and sectors has emerged as a main priority for Europe. Common European Data Spaces (CEDS) aim to make this possible in a trusted, secure, and interoperable way. However, setting up such environments remains a complex task, especially for public sector actors with limited resources. This paper presents the first steps toward a Minimum Viable Dataspace (MVD) for the European public sector. The goal is to support secure data sharing among authorized entities, in alignment with key European dataspace initiatives. The MVD architecture integrates essential components: a Dynamic Attribute Provisioning Service (DAPS), a data exchange agent that enables data sharing and storing, and multiple open-source dataspace connector software blocks deployed in real pilot environments. The setup includes components for partial usage control, authentication, and policy enforcement. Thus, it demonstrates a controlled framework for data exchange among authorized participants. Early pilot feedback confirms the usability and readiness of the MVD. This implementation can lay the foundation for further iterations, aiming for easier and scalable integration with wider European data ecosystems.

## 1 Introduction

As Europe advances its vision for a unified digital ecosystem, trusted and sovereign data sharing across sectors and borders has taken center stage. The European Commission’s strategy for Common European Data Spaces (CEDS) [1] calls for interoperable infrastructures that support transparency, data protection, and self-determination [2], [3]. These values are fundamental and deeply rooted in the EU’s digital agenda [4]. Yet, building such environments remains a considerable challenge for many public sector organizations. These institutions often lack the technical capacity and hardware resources to engage in complex, large-scale dataspace efforts [5], [6].

To address this gap, the concept of a Minimum Viable Dataspace (MVD) has emerged [7]. It offers a lightweight yet functional alternative: A small-scale, operational environment that enables secure data exchange among verified participants. While not a full dataspace, the MVD is designed to be aligned with key principles of broader European initiatives, such as the International Data Spaces Association (IDSA) [8], GAIA-X [9], and EOSC [10]. It acts as a stepping stone, meaning that it is small enough to be deployed and tested in real conditions, but robust enough to be interoperable with future or existing data ecosystems.

In this work, a user-friendly implementation of such an MVD is presented, tailored to the needs of the European public sector. Up to the time of this study, the initiatives that fall under the umbrella of the CEDS’ public administration sector are the European Legal Data Space (ELDS) [11], the Once Only Technical System (OOTS) [12], and the Public Procurement Data Space (PPDS) [13]. The proposed MVD is designed in the context of future integration with PPDS, co-existing with other dataspace implementations that will be part of the public procurement sub-domain.

The motivation for proposing this implementation is straightforward. There is a need for a clear, open-source guideline on how to deploy an MVD. Such a model can lower the complexity of first steps and speed up the adoption of the dataspace concept. In the short term, it offers a pathway towards realizing public sector initiatives such as the aforementioned ones. In the longer term, because the deployment is generic, it can support faster adoption of data spaces across other CEDS domains as well. The proposed approach draws from real-world scenarios, with three pilot deployments conducted in a proper distributed manner.

Importantly, this paper does not introduce another abstract framework. It presents a working model, tested with real datasets and live connector software instances. As outlined, the implementation is open, replicable, and aligned with European priorities on controled data sharing and interoperability. More than a proof of concept, it can serve as a practical guideline for deploying an MVD. The approach is incremental and realistic, helping public actors lower the entry barrier and take part in shaping Europe’s digital CEDS landscape. Recent research continues to highlight the need for open-source, modular, and extensible data spaces that can evolve with regulation and policy. The proposed MVD framework supports that vision.

The rest of this paper is structured as follows. Section Literature Review (2) discusses related work on data spaces and existing approaches. Section Framework Description (3), introduces the proposed framework for an easily deployable and open-source MVD for the public sector. Section Prototype Deployment (4) presents a structured deployment plan for the MVD, acting as a deployment guideline, along with its initial assessment. Section Initial Testing and Evaluation (5) presents an initial test of the proposed MVD, as well as an early assessment on the findings and their implications. Finally, Section Conclusion (6) closes the paper and outlines next steps.

## 2 Literature Review

Data spaces are a cornerstone of the European Strategy for Data, aiming to enable interoperable and sovereign data sharing across sectors and borders [14], [15]. The legislative foundation enabling such trusted data exchange is defined by the Data Governance Act (DGA) [16], which introduces trusted data intermediaries and mechanisms for data altruism, and the Data Act [17], which establishes horizontal rules for fair access and use of industrial data, including portability, cloud switching and contractual fairness. The European Commission’s latest progress report on CEDS highlights ongoing deployment across domains, including public administration and public procurement, and reinforces the need for reusable building blocks and reference architectures [18]. Complementing this, the Data Spaces Support Centre (DSSC) Blueprint consolidates business, legal, governance and technical guidance into a modular set of dataspace components, including identity, policy and interoperability services [19], [20].

Technically and architecturally, the IDSA provides foundational work on data sovereignty. The IDS Reference Architecture Model (RAM) defines roles, trust anchors, identity services, and usage-control concepts [21], while the IDS Rulebook describes the operational and legal framework for participation in a dataspace [22]. The Dataspace Protocol (DSP), jointly developed by IDSA and the Eclipse Foundation, standardizes connector interactions for onboarding, catalog discovery, contract negotiation, and policy binding [23]. These specifications collectively represent the most widely adopted blueprint for sovereign data exchange in Europe.

In software implementations, the Eclipse Dataspace Components (EDC) project has emerged as the most widely adopted open-source connector framework for building dataspace nodes [24], [25]. EDC provides modular components for identity, control and data planes, and supports deployment across cloud and on-premises environments. Sovity extends EDC by offering a connector distribution with ready-to-use operational tooling (portal/UI, policy management, automated provisioning), reducing deployment complexity for newcomers [26], [27]. These EDC-based implementations support machine-readable usage policies aligned with IDSA usage control guidelines [28]. Recent research advances usage control enforcement, enabling continuous and dynamic policy verification in distributed scenarios [29].

Scientific research on data spaces has accelerated in recent years. In [30] a comprehensive systematic survey on data spaces is provided, reviewing technical architectures, software maturity and research gaps. Moreover, in [31] dataspace design options are classified along organizational, governance, and architectural dimensions, offering a structured framework for practitioners. Furthermore, data spaces as meta-organizations are conceptualized in [32], emphasizing governance coordination over purely technical interoperability. Other studies focus on sector-specific implementations. For example, [33] investigate governance building blocks in data spaces, while a review on data spaces in manufacturing, reporting lessons from industrial ecosystems is presented in [34].

Interoperability (especially catalog interoperability) remains a persistent challenge in federated ecosystems. How federated data catalogs can facilitate dataset discovery while maintaining sovereignty is demonstrated in [35]. An approach based on the Eclipse XFSC component is introduced in [36]. These findings align with the DSSC Blueprint, stressing modular, interoperable building blocks.

Several large-scale European initiatives demonstrate that data spaces are no longer theoretical. Catena-X operationalizes sovereign data exchange in the automotive sector using EDC and DSP-based components [37], [38]. The Mobility Data Space (MDS) enables exchange of mobility datasets between heterogeneous actors while maintaining sovereignty [39], [40], [41]. The Data Space for Cultural Heritage [42] and the European Open Science Cloud (EOSC) [43], [44] demonstrate dataspace concepts in culture and research domains. Of particular relevance to this work (as previously outlined), the Public Procurement Data Space (PPDS) aims to federate national procurement datasets with TED to improve transparency, interoperability and analytics capacities across the EU [45], [46], [47], [48].

Despite these advances, practical deployment remains challenging for public administrations, which often lack the technical capacity to deploy complex infrastructure or implement advanced policy management. Recent surveys highlight that a major gap persists between dataspace conceptual frameworks and minimal, operational deployment models [30], [31]. This motivates the contribution of this work: the definition and implementation of a MVD using open-source components (EDC-based connector, Keycloak-based DAPS, and a lightweight Data Exchange Agent). The proposed MVD provides the smallest possible operational configuration that supports secure, policy-driven data exchange and can act as a stepping stone toward full participation in Common European Data Spaces.

## 3 Framework Description

### 3.1 Architecture and components presentation

The proposed MVD setup aims to target technical viability, instead of than full production maturity. It is defined through four essential steps. The first is the establishment of a DAPS, in order to enable secure identity management. The second is the deployment of at least two remote connectors. The third step is a successful DAPS-based authorization and authentication between the connectors. The fourth and final step is demonstrated data exchange between the aforementioned connectors. This scope helps establish proof-of-concept functionality for sovereign data sharing. At the same time, it recognizes limits in governance structures and scalability, which could be tackled in a broader dataspace environment with the addition of new software components.

The current MVD framework has been designed to support distributed operation beyond local or simulated setups. It is fully operable across different machines, with each machine running its own dataspace connector. Each participant engages in controlled, policy-driven data exchange. The architecture of the MVD is built around three main components:

• Dynamic Attribute Provisioning Service (DAPS): Positioned at the core of the architecture, it provides secure identity and access management as the foundation of trust in the dataspace.
• Dataspace Connector: An open-source software, it enables access to the dataspace and ensures easy and seamless deployment across participants. It aligns with IDSA specifications and supports partial usage control out-of-the-box.
• Data Exchange Agent: It complements the connector by enabling actual data exchange for each dataspace participant, allowing them to act both as consumers and providers.

**Fig. 1:**
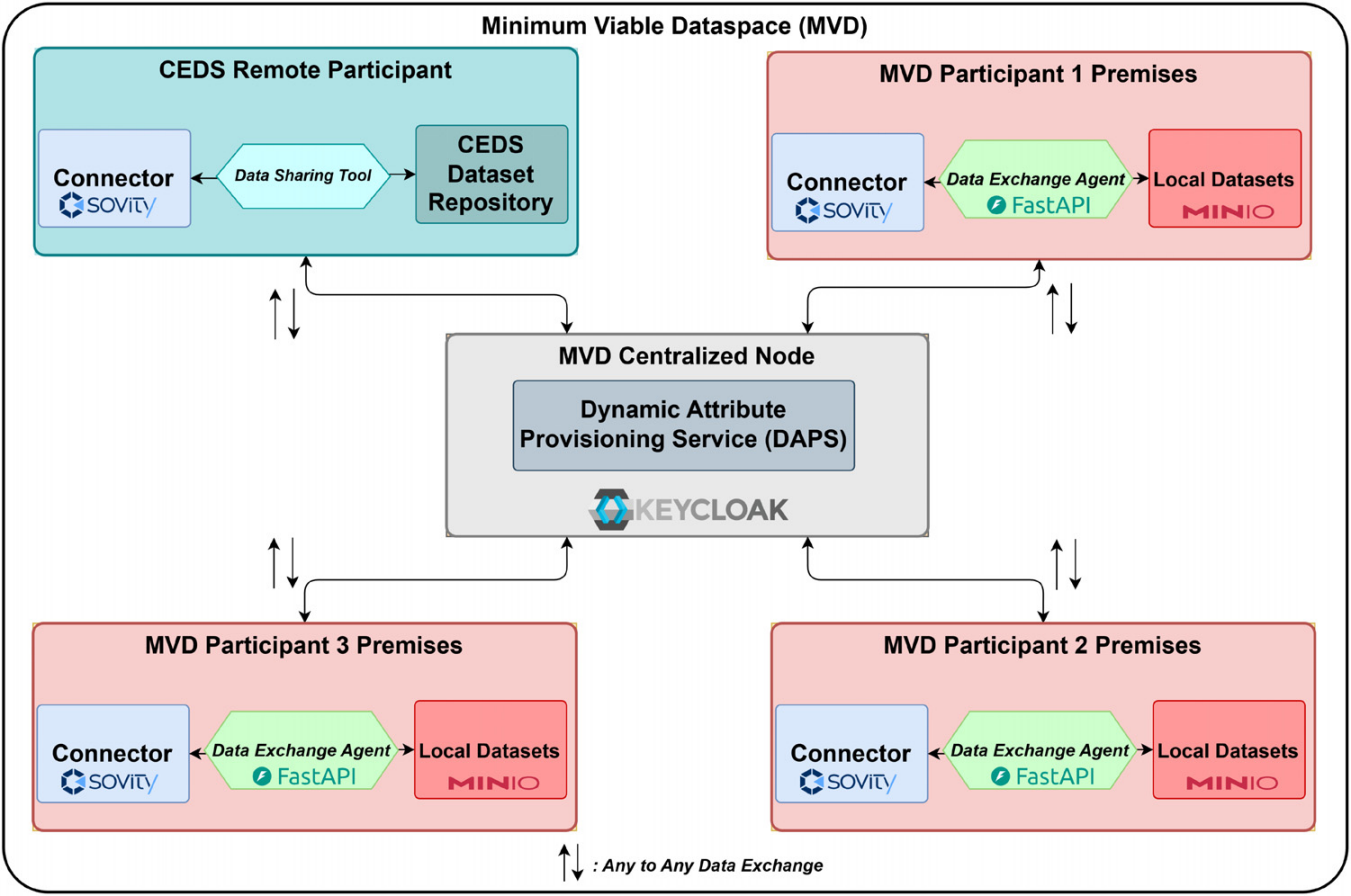
The architectural overview of the proposed Minimum Viable Dataspace (MVD).

Together, these components form the outlined practical and replicable MVD setup that demonstrates distributed, policy-driven data sharing in action. The system’s architectural overview is presented in Fig. 1. The diagram shows a centralized node, where DAPS is deployed. This node manages the authorization and authentication rights of all dataspace participants. In the proposed setup, DAPS runs together with an NGINX-based reverse proxy. NGINX [49] handles the incoming requests and directs them to DAPS. With Certbot [50], HTTPS is enabled by issuing and renewing SSL certificates automatically. This way, all communication with DAPS is secure and reliable.

As for the Connector and Data Exchange Agent instances, these are then deployed at the premises of three remote participants. Each Data Exchange Agent communicates with a predefined storage tool to enable data forwarding and retrieval. By default, the Agent software automatically deploys a MinIO [51] object storage, but this can be modified according to user needs. As illustrated in the figure, remote participants already involved in other CEDS Data Spaces can also join the proposed MVD. Once authorized through DAPS, they can act as both consumers and providers within the MVD. This containerized setup enables public organizations to create and test operational data spaces without having to build them from scratch. As previously outlined is deployable on-premises or in the cloud, and supports integration with broader federated ecosystems. Moreover, the previously noted DAPS authentication and basic policy definition capabilities allow each connector to support partial access control. After contract negotiation, the system binds all defined policies during data transfer. Post-access usage control enforcement remains a target for future iterations.

The use of Keycloak for DAPS was made because it provides dataspace administrators with strong control, flexibility, and open-standards support. Keycloak supports multiple protocols, such as OpenID Connect, OAuth2, and SAML. It allows to use service accounts, custom mappers (e.g. DAT Mapper) and fine-grained settings. Because it is open source, there are no licensing fees. Moreover, administrators can host, configure, and tailor it based on their needs. Compared to proprietary Identity and Access Management systems (IAMs) like Auth0 [52] or Okta [53], Keycloak avoids vendor lock-in. From open source alternatives like Gluu [54] or FusionAuth [55], Keycloak has a larger user and developer community. That can help when administrators seek to find help, discover plugins, or keep the software maintained.

In short, Keycloak fits the DAPS vision well because it gives control over data, deployment, and custom rules. It balances power with openness in ways many commercial and open-source options do not. It should be noted that a centralized DAPS suits MVD testing, but creates a single point of failure. Production systems require resilience through multiple measures. A future iteration should implement redundancy through DAPS clustering or distributed trust anchors. A high-availability configuration, along with concrete failover mechanisms should also be established, in order to enhance resilience for production environments. The current DAPS component is deployed using Docker restart policies to ensure automatic recovery in case of failure or host restart. In addition, health checks are defined to monitor its operational state, in order to trigger restarts when the service becomes unresponsive. These initial measures should be expanded in a production version of the MVD.

For the connector software, the final choice is sovity’s Connector, an open-source tool based on the Eclipse Dataspace Components (EDC) Connector [56], extended and maintained under the sovity initiative [57]. The main reason behind its selection is because it is built on the EDC core, which ensures strong compatibility and avoids replication of existing solutions. Sovity adds useful extensions for easier deployment, management, and usability. It reaches a high Technology Readiness Level (TRL) in practice in dataspace projects (inherent from the EDC’s software), which gives confidence in its stability [58]. The implementation uses exclusively the open-source community edition of the sovity connector, without proprietary extensions. All components and configurations remain fully accessible through sovity’s public repositories.

By comparison, the FIWARE TRUE Connector [59] (as well as with its extensions) is often seen as an advanced connector too, and it is included in discussions of mature dataspace technologies. Still, sovity/EDC has been selected over TRUE, because EDC already shows higher readiness and broader experimental use. TRUE is less mature in deployments so far, and fewer real-world projects seem to rely on it [58]. Because EDC (and sovity’s edition) is more widely used, it gives more confidence in stability and support [60]. Other connector software implementations also exist (mainly various custom-built implementations), but they often lack the open-source nature, community support, extensions, or maturity of EDC-based solutions. Using the sovity’s EDC extension gives a balanced path: Leveraging EDC’s standards and community, and adds usability, stability, and readiness.

As for the Data Exchange Agent, this complementary software tool is based on FastAPI [61], an API building web-framework. As previously outlined, the Agent handles data forwarding and retrieval to/from a MinIO object store (by default), though users can replace that with another object storage if needed. It complements the dataspace connector by enabling each participant to both provide and consume data. By default, the Agent deploys a MinIO instance. As explained, the Agent aims to provide simplified data transfer and storage integration. Therefore, it prioritizes simplicity of the deployment and transparency of the data flows, over the native EDC data plane’s advanced features. The design helps to improve modularity and reproducibility for proof-of-concept testing, while acknowledging limitations in protocol abstraction and scalability (which could be addressed with the adoption of EDC’s data plane).

FastAPI is a solid choice because it is fast, simple, and supports concurrency. Based on benchmarks allows for many requests at once, with low overhead [62], [63]. It also gives automatic documentation and type checking, which helps in development and in catching errors early. For many API-based data systems, responsiveness and reliability are important. FastAPI meets those demands. Thus, it has been selected over other similar frameworks.

Regarding MinIO, it is chosen as a default storage option, mainly because it is S3-compatible, lightweight, and performs well. It scales easily and works on commodity hardware, among other advantages [64]. It is already used in production in many settings, including cloud, edge, and on-premises deployments [65]. Having MinIO as the default means that users can expect lower friction for adoption, stable behavior, and known patterns for integration.

### 3.2 Applicability in the CEDS public sector

A Minimum Viable Dataspace in the public administration sector offers a focused and realistic step toward realizing the goals of the Common European Data Spaces. The use of a connector that complies with several EU initiatives (sovity’s EDC-based connector) ensures alignment with the evolving European dataspace architecture. It also helps keep the initial deployment lightweight and functional. The MVD demonstrates that even a minimal setup can enable controlled data exchange between public actors. This is important because most public administrations face technical and organizational barriers that make full dataspace adoption difficult. A minimal, working deployment lowers the entry barrier and proves the feasibility of the dataspace concept in real administrative contexts.

Within the ’Public Administration’ sector of CEDS, the MVD aims to support cross-agency interoperability and the secure exchange of sensitive information. Data in this sector is often isolated. This is mainly due to differing legal mandates, legacy systems, and security requirements. The MVD addresses this by providing a standards-based environment intended to support controlled data sharing among the participants. This promotes trust among institutions and encourages collaboration on shared challenges such as digital governance or policy evaluation. Since it integrates intial data usage control and identity management, the MVD aims to support traceable and policy-controlled access through the implemented connector and DAPS setup. Formal legal compliance and governance validation are outside the scope of this implementation.

The public procurement subdomain stands to gain substantial benefits from this approach. Procurement involves the exchange of structured and semi-structured data across multiple actors and jurisdictions. The MVD can serve as a testing ground for transparent and verifiable data flows between entities involved in the procurement subdomain (e.g., contracting authorities, suppliers, oversight bodies etc). It can support automated validation of tender information, performance tracking of awarded contracts, and publication of procurement data in machine-readable formats. Such capabilities can enhance transparency and reduce administrative overhead. As previously outlined, in the long term, the MVD could be federated with the Public Procurement Data Space (PPDS) or act as a representative node, linking national or regional procurement datasets with the broader European infrastructure. This would ensure that even small-scale (or experimental) deployments contribute to the formation of a coherent, interoperable, and trustworthy public procurement dataspace ecosystem.

## 4 Prototype Deployment

A dataspace is only as strong as the trust and reliability between its participants. The DAPS and connector play central roles in this minimum viable structure. DAPS acts as the entry point for authenticating and authorizing connectors. The latter, once approved by DAPS, allows organizations to share and request data. Deploying MVD’s DAPS and connector instances does not need to be complex, but it does require following a clear sequence of steps.

### 4.1 Deploying a dataspace connector

The first step is to establish trust. Every connector must prove its identity to the dataspace and to the DAPS (also see Section 4.2). This trust relationship is achieved through digital certificates and secure keys. These certificates must be generated with the right properties so that the connector can both present itself and verify others. Each connector should be accompanied by a certificate that links back to a trusted root authority. In practice, this means that before anything runs, a security expert from each organization that wishes to enter the dataspace must prepare a small set of certificates and keys that will serve as the basis for secure communication.

Once trust anchors are in place, the second step is the preparation of the connector environment. At this point, the service is not yet operational. The focus is on setting up the connector’s configuration so that it “knows” how to behave once started. This includes assigning a unique participant identifier, pointing the connector to the correct DAPS service, and listing the catalog addresses of other connectors (if they are known). Alternatively, a reference to a Federated Catalog is sufficient, as this service manages the assets of all MVD connectors. The participant identifier is important: it does not have to be complicated, but it must be unique inside the dataspace. It is also an essential value for the previous step of certificate generation. Preparing these values in advance ensures that the connector will not clash with others and can be recognized as a distinct participant.

The third step is the deployment of the connector. At this stage, the connector is launched, typically within a Docker container. The aim is to make it operational in a way that is stable and repeatable. What matters from a deployment perspective is not the specific orchestration tool, but the discipline of keeping configuration parameters well-documented. Environment-specific values, such as addresses or identifiers, should not be hard-coded. Doing so enables the connector to be replicated, migrated, or upgraded with minimal friction. Based on this approach, the connector software of the proposed MVD is easily replicated across three tested pilots. Each pilot’s security expert runs the process of issuing the outlined certificates, aiming for a seamless integration with DAPS.

The fourth and final step is validation. Once the connector is operational, it will normally expose a user interface (UI), along with service endpoints. These allow end-users to verify that the connector has started correctly, that it shows the right participant identity, and that it can communicate securely with others. In this phase, organization end-users should perform simple tests to ensure its smooth operation. They can do so by logging into the interface, confirming that the connector is registered to DAPS, and ensuring that it can connect to at least one other participant, allowing for potential data exchange.

To summarize, deploying the dataspace connector requires organizations to follow four main stages:

1. Establish digital trust through the controlled generation and use of certificates.
2. Prepare the connector’s configuration, including its unique identity and connection details.
3. Deploy the connector in a containerized environment with clean and portable settings.
4. Validate secure and seamless operation through the connector’s UI and service endpoints.

### 4.2 Setting up DAPS and enrolling connectors

The starting point is deploying the software. DAPS is mainly packaged as a containerized service, which simplifies setup and operation. Before enabling the package, the environment must be prepared with the proper hostname and access credentials. A valid DNS hostname is essential because DAPS must be reachable from outside the local machine. Administrators should also define passwords, as well as additional security-related parameters in the configuration files. Once this preparation is complete, the DAPS service can be safely started. Then, it is accessible through its web-based administration UI.

The second phase is enabling secure communication. DAPS must always operate over HTTPS, which requires valid SSL certificates. For this, a web server (e.g., NGINX) can be placed in front of the service. Administrators configure the server to use certificates obtained through Certbot, which are then linked to the DNS hostname of the DAPS machine. Once applied, the DAPS interface is reachable through a secure HTTPS connection, and participants can trust the identity of the service.

The third step is configuring DAPS to interact with the existing dataspace connectors. In practice, this means creating a dedicated realm for a dataspace use. Then, the administrators proceed with defining the rules that describe how connectors authenticate themselves. Each connector is registered as a client, using values that match the certificates created during the connector’s setup (see Section 4.1 above). The client configuration defines what claims and attributes each connector presents, and how these are validated by DAPS. The participant identifier set in the connector’s configuration must also be mirrored here, ensuring that the two software components are aligned.

Once participants are added, DAPS begins to actively engage as an authorization service. Connectors that are correctly registered can request and exchange data. Those that are not registered will fail to authenticate and cannot see or access any assets. A simple way to test this is by deploying two connectors: One registered with DAPS and one without. If the unregistered connector cannot access the registered one’s catalog, while the registered connector can, this demonstrates that the authorization mechanism works. This forms the basis of an MVD.

In summary, deploying a Keycloak-based DAPS involves four main steps:

1. Deploy the DAPS service with a valid DNS hostname, along with secure configuration.
2. Enable HTTPS using a reverse proxy and SSL certificates.
3. Configure DAPS realms and enroll existing connectors as clients with proper attributes.
4. Validate the setup through controlled testing with one or more connectors.

### 4.3 Enabling the data exchange agent

As previously explained, the Data Exchange Agent is designed around a Fast API web service running inside a Docker container. It is a lightweight tool that acts as a bridge between storage backends and the dataspace connectors. In this case, the storage backend is MinIO, which serves as the agent’s internal repository. There, datasets are stored, retrieved, and managed. As already outlined, MinIO is used as the default storage option for simplicity and compatibility, but this choice can be changed based on user needs or existing infrastructure.

The first step in deploying the agent is preparing the environment. The agent and its storage system are both defined through a Docker Compose configuration, which describes how the services will start and connect to each other. This setup allows the entire solution – the Fast API service and the MinIO storage – to be launched with a single command. Before deployment, administrators may adjust configuration parameters, such as the access credentials or the names of the storage buckets where data will be kept.

Once started, the agent exposes two main endpoints that enable data movement in and out of the dataspace. The first endpoint provides a way to forward tabluar data for sharing. When called, it reads a selected file from the storage backend and returns it in a structured format. The second endpoint works in the opposite direction. It accepts data in structured format, and stores it in the designated bucket within MinIO. This approach allows dataspace connectors / participants to easily share available datasets. Thus, through these two endpoints, the agent supports both data offering and data receiving operations in a standardized, format-neutral way.

To integrate with a dataspace connector, the agent’s endpoints are registered as REST-based data sources and sinks. For example, when sharing an asset in the dataspace, the sending endpoint contributes to the connector’s “Data Offer”. At the same time, after successfully negotiating a data contract, the receiving endpoint enables the connector’s “Data Sink”, in order to download the data. This setup allows connectors to transfer data directly through HTTP interactions, without the need for complex middleware. The web-based interface of MinIO provides a convenient way to inspect the stored datasets. Administrators can view, upload, or download files directly from the browser, making it easy to verify whether transfers were successful. It is also possible to extend this setup – for example, by scaling the service across multiple instances to handle larger datasets.

In summary, deploying the data exchange agent involves three straightforward steps:

1. Prepare the containerized environment using Docker Compose, defining both the agent and its storage service.
2. Launch the services and verify that the agent is reachable and connected to the storage backend.
3. Register the agent’s endpoints with the dataspace connector to enable secure, bidirectional data exchange.

## 5 Initial Testing and Evaluation

### 5.1 Data exchange sequence flow

**Fig. 2:**
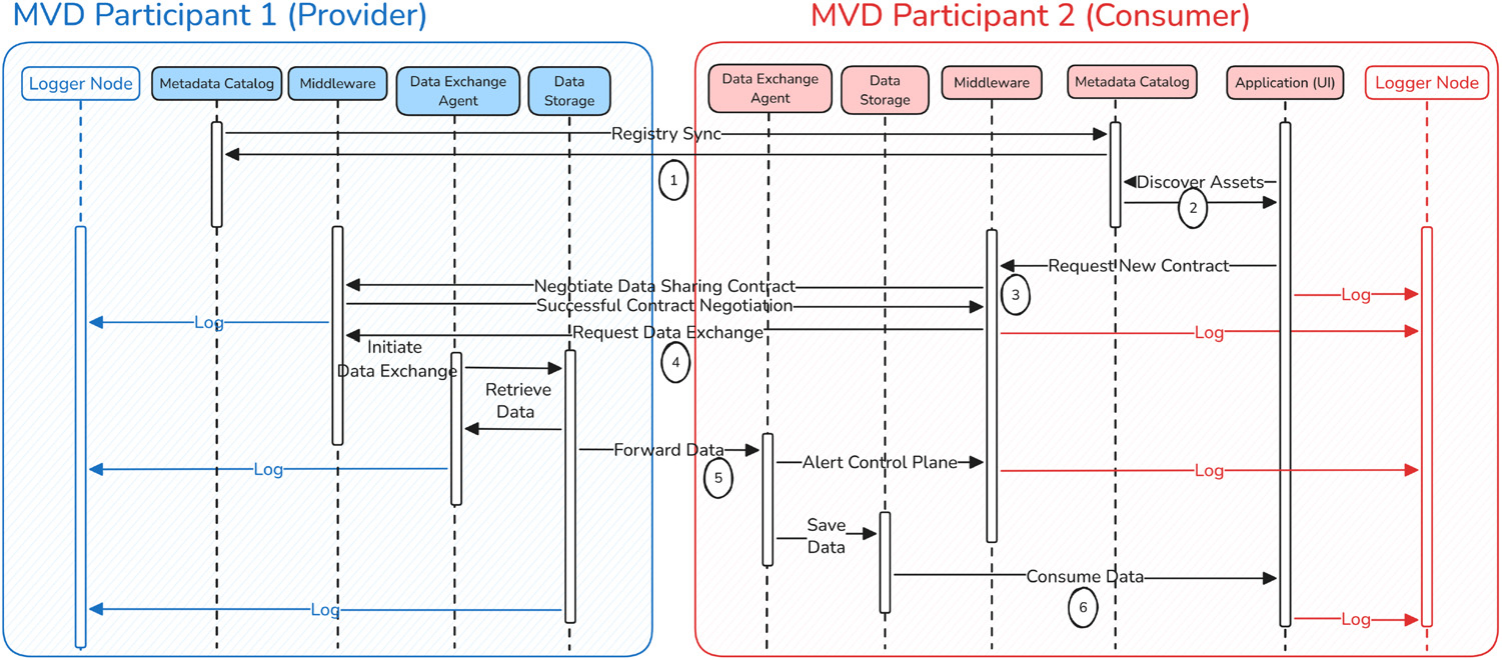
A sequence diagram presenting the data exchange process between two dataspace participants.

Fig. 2 presents the sequence flow of a data exchange process between two MVD participants.

The process is divided into six main steps, detailed as follows:

1. All dataspace participants first receive metadata about the available assets within the MVD. This is being achieved through their Metadata Catalog synchronization. The catalog of each participant (part of the connector software) collects information on all available assets and redistributes it to all other MVD participants.
2. End-users can then browse and view assets through their connector’s UI. They can explore all available offerings and optionally select one for contract negotiation.
3. If the end-user decides to consume a specific asset, they initiate the contract negotiation phase by submitting a contract request. This request is sent to the connector’s Middleware and then forwarded to the potential provider’s Middleware.
4. When the contract negotiation is successfully completed, the data exchange process can start automatically or later, depending on the end-user’s choice.
5. The provider’s middleware receives the data exchange request and communicates with its Data Exchange Agent. The Agent then retrieves the requested data asset from the storage system (in this case, MinIO).
6. After retrieving the data asset, the provider’s Data Exchange Agent transfers it to the consumer’s Agent, which notifies its middleware upon receipt. The data are stored in the consumer’s storage system and become available for use by the end-user.
7. Finally, the consumer accesses and uses the received data asset.

It should be noted that information about each step is stored as local logs by every participant.

### 5.2 System testing and early assessment

All three connectors, as well as the centralized DAPS, run on identical physical machines to keep the setup consistent. The machines are equipped with 4 vCPUs based on AMD EPYC 2nd Generation processors, 8 GB of RAM, and 160 GB of SSD storage. All systems run Ubuntu 22.04 and share the same configurations. This uniform setup helps ensure that testing conditions are equal across the Italian, Slovenian, and Ukrainian deployments. The same holds for the dedicated deployment of DAPS. It should be noted that some parts of the screenshots have been intentionally obscured for security and privacy reasons. These hidden elements include sensitive identifiers, URLs, or access tokens that could expose internal configurations (or credentials). The modifications do not affect the interpretation of the presented results.

**Fig. 3:**
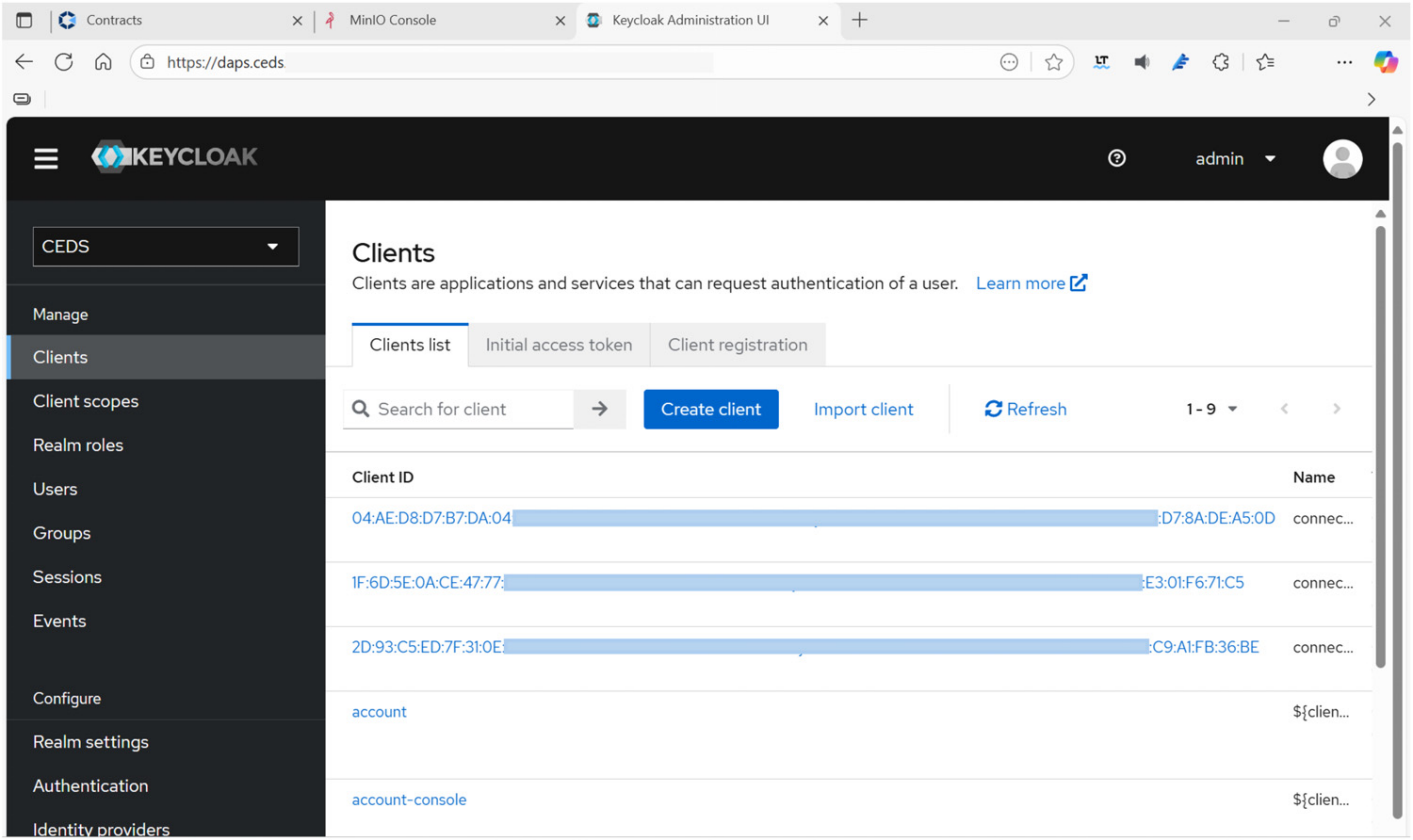
Screenshot from Keycloak DAPS operating.

The testing scenario starts by confirming that the Keycloak-based DAPS is active and operational (Fig. 3). Three dataspace connectors are already registered, representing the Italian, Slovenian, and Ukrainian public procurement entities. Each connector is enrolled, authenticated, and authorized through DAPS. As long as DAPS remains in control, the dataspace stays secure. Any new participant must first go through the same process of authorization and authentication. Only then can it access the dataspace, view existing data offers, publish its own, and engage in data exchange or contract creation with other participants.

The next phase of the scenario focuses on the local preparation of data sharing. The Italian public procurement entity decides to share a specific tabular metadata file with its Slovenian and Ukrainian counterparts. The file, a CSV dataset, contains information about suppliers’ participation across public tenders. Each row represents a Common Procurement Vocabulary (CPV) category. It lists the minimum, maximum, and average number of suppliers per tender item. This simple dataset can help other dataspace participants understand how companies in Italy behave in different procurement categories. It can be particularly useful when there is limited historical data, offering a reference point for market activity in specific tender areas.

**Fig. 4:**
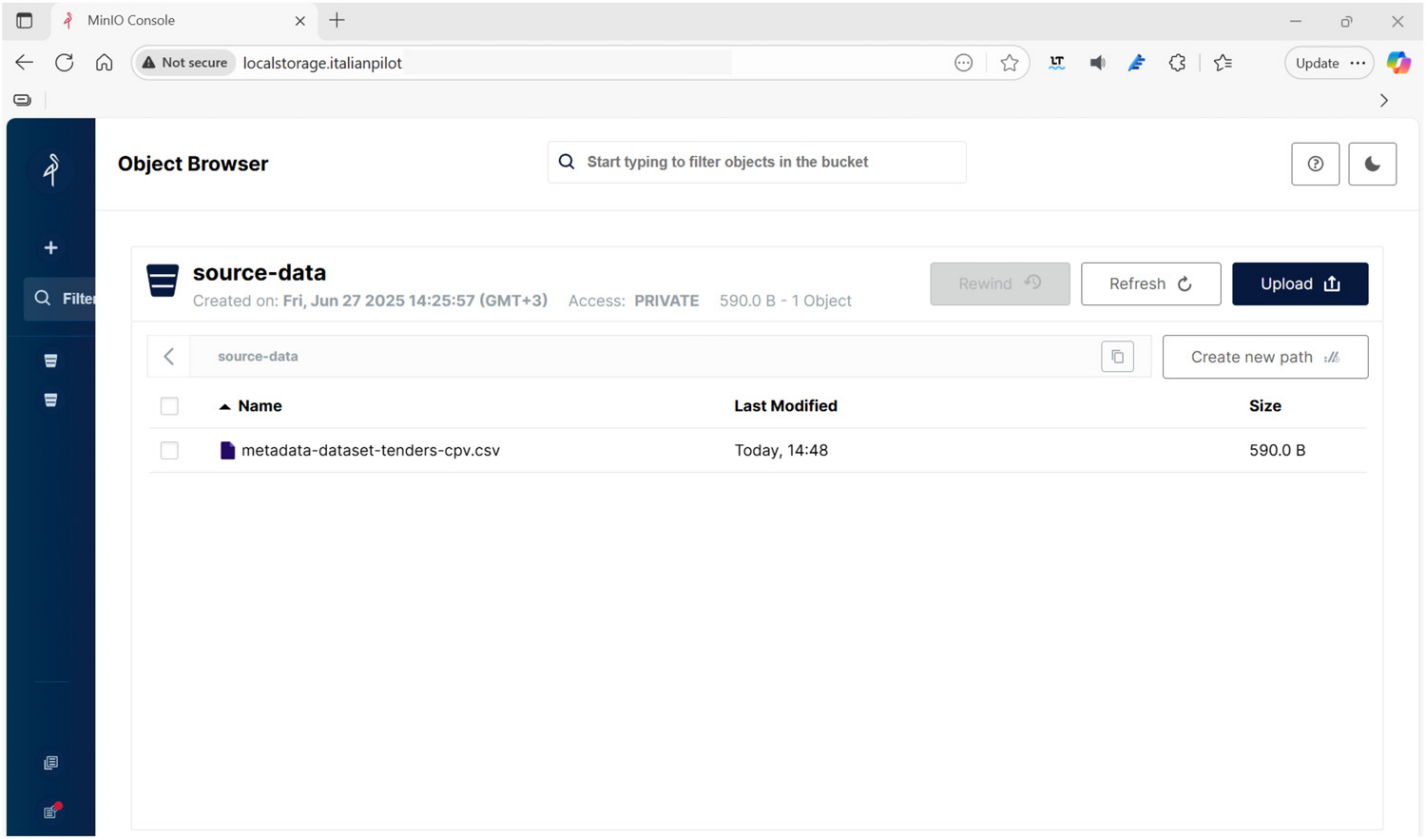
Snapshot from Italian participant’s MinIO Source Data Bucket.

The process begins with the Italian participant’s end-user accessing the local MinIO storage (Fig. 4). Through the interface, the user uploads the CSV file to the designated data bucket, named “source-data”. This action marks the first step in data preparation and availability within the dataspace. The file is now stored locally, ready to be exposed through the connector for controlled sharing with the Slovenian and Ukrainian participants.

**Fig. 5:**
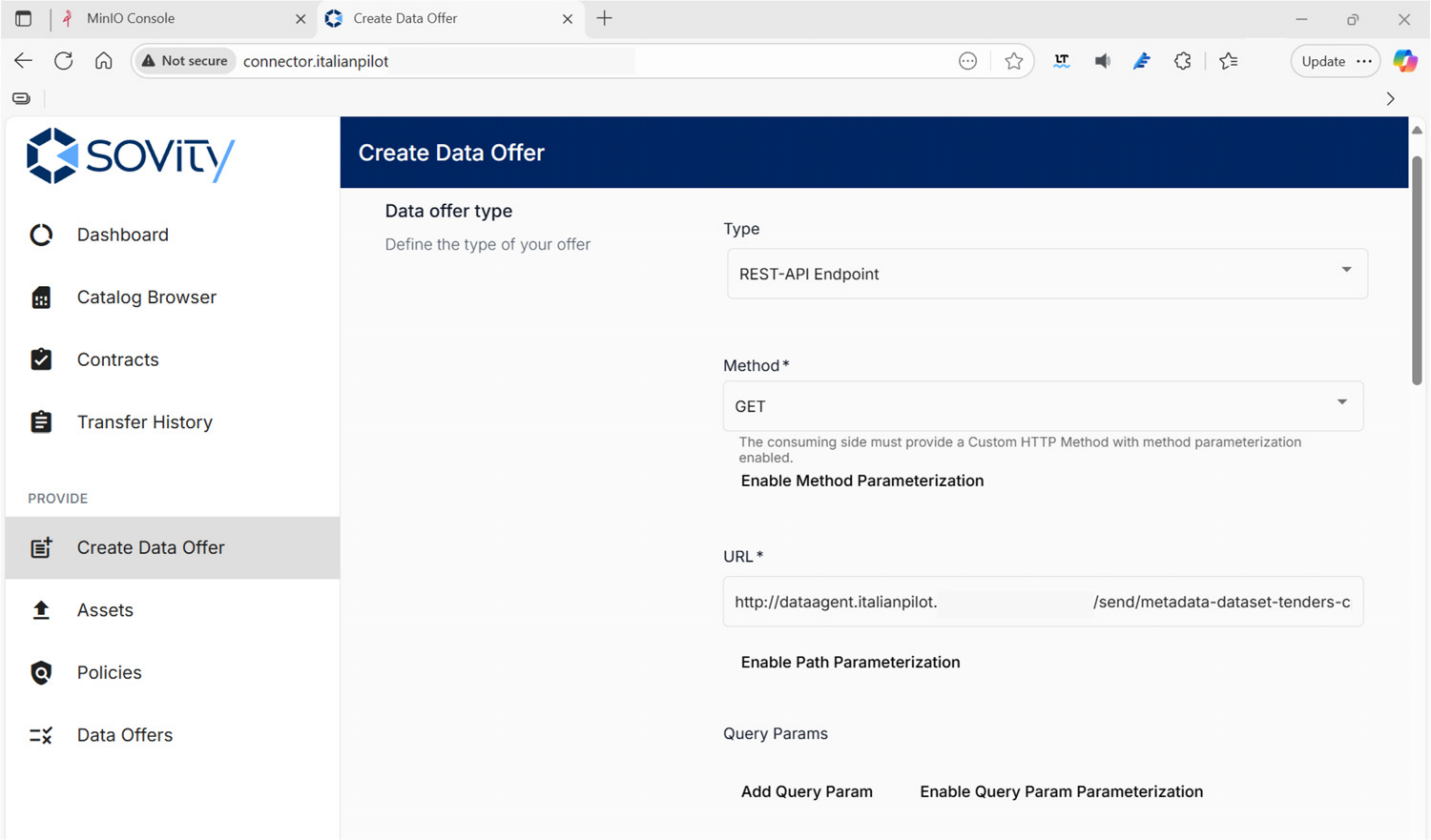
Creating a Data Offer by the Italian public procurement entity.

In the second phase, the Italian public procurement end-user moves from local preparation to actual data sharing. They open the dataspace connector’s user interface, which is deployed within the Italian entity’s infrastructure. The same setup exists for the Slovenian and Ukrainian participants. Through the UI, the Italian end-user starts creating a new data offer (Fig. 5). In the offer’s configuration, the endpoint field requires the local Data Exchange Agent’s URL. At the end of this URL, the user adds the filename of the CSV previously uploaded to MinIO, following the structure http://local-data-exchange-agent-url/send/<filename>. This step links the published offer directly to the stored dataset.

**Fig. 6:**
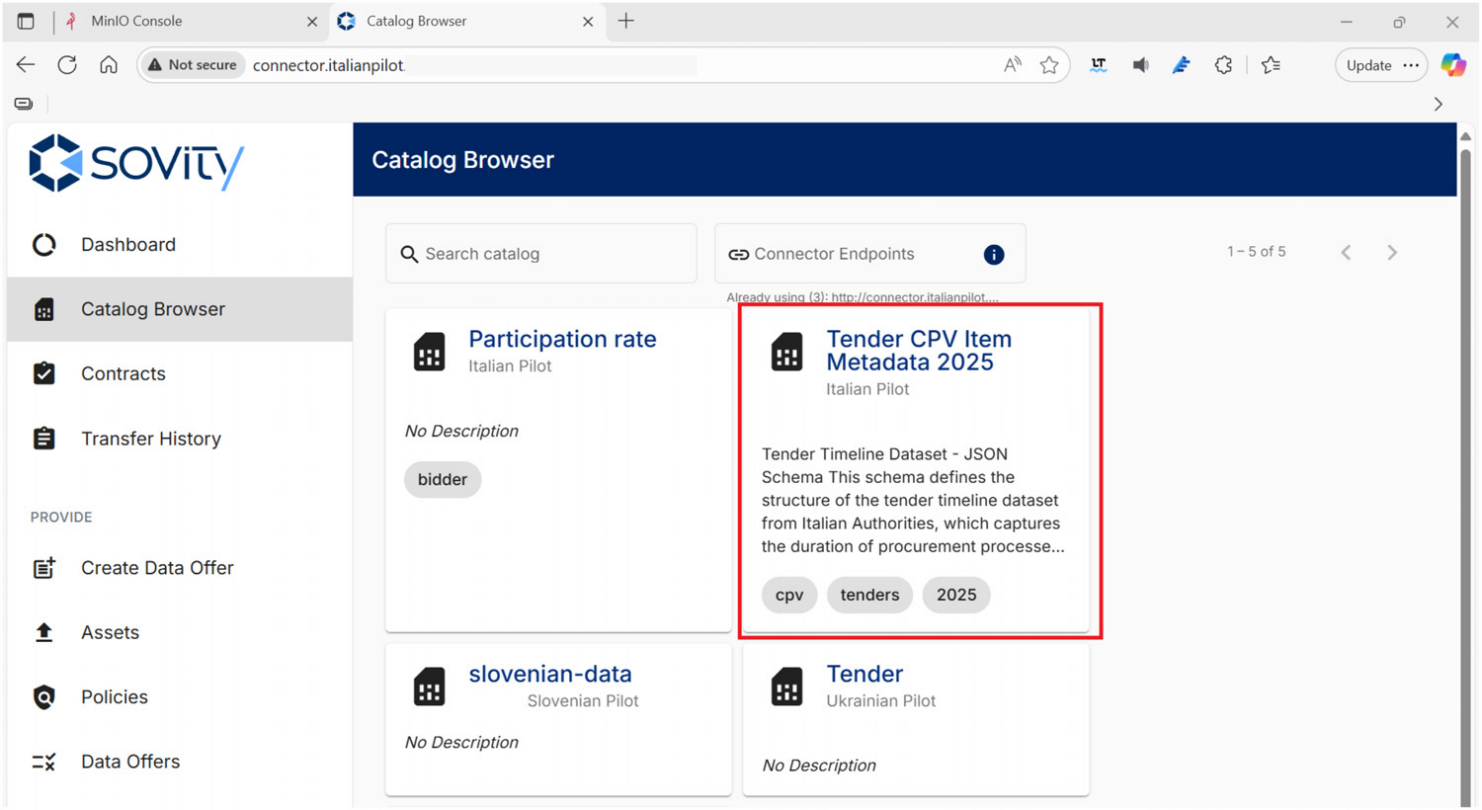
Screenshot from the Italian participant’s Catalog Browser view.

Once all required fields (such as name, ID, and description) are completed, the user can define access conditions. The system allows offers to be published either with usage policies or as open assets. For this test, the Italian end-user publishes the dataset without restrictions. The asset becomes visible in the dataspace’s shared catalog. With a visit to the “Catalog Browser” section (Fig. 6), the user can now view all active data offers across the dataspace, including the new CSV dataset they have just published.

**Fig. 7:**
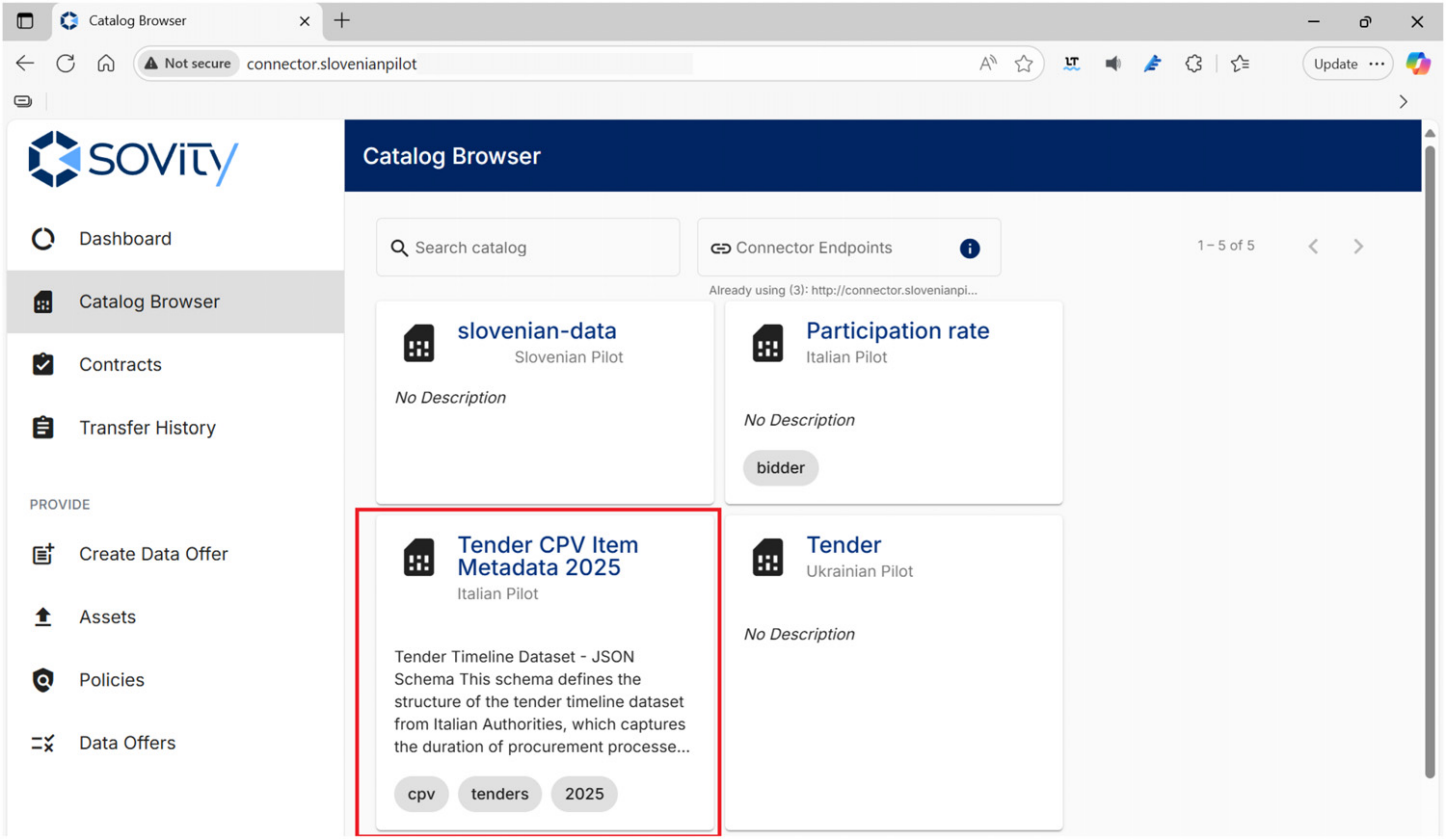
Screenshot from the Slovenian participant’s Catalog Browser view.

In the next phase, the focus shifts to data consumption. The Slovenian public procurement entity initiates the process to consume the dataset shared by the Italian counterpart. The Slovenian end-user connects to their local connector UI, deployed within their organization’s premises. From there, they open the “Catalog Browser” tab (Fig. 7) and view all available data offers in the dataspace. Among them, they locate the newly published CSV dataset from the Italian public procurement entity. They proceed to start a contract negotiation.

**Fig. 8:**
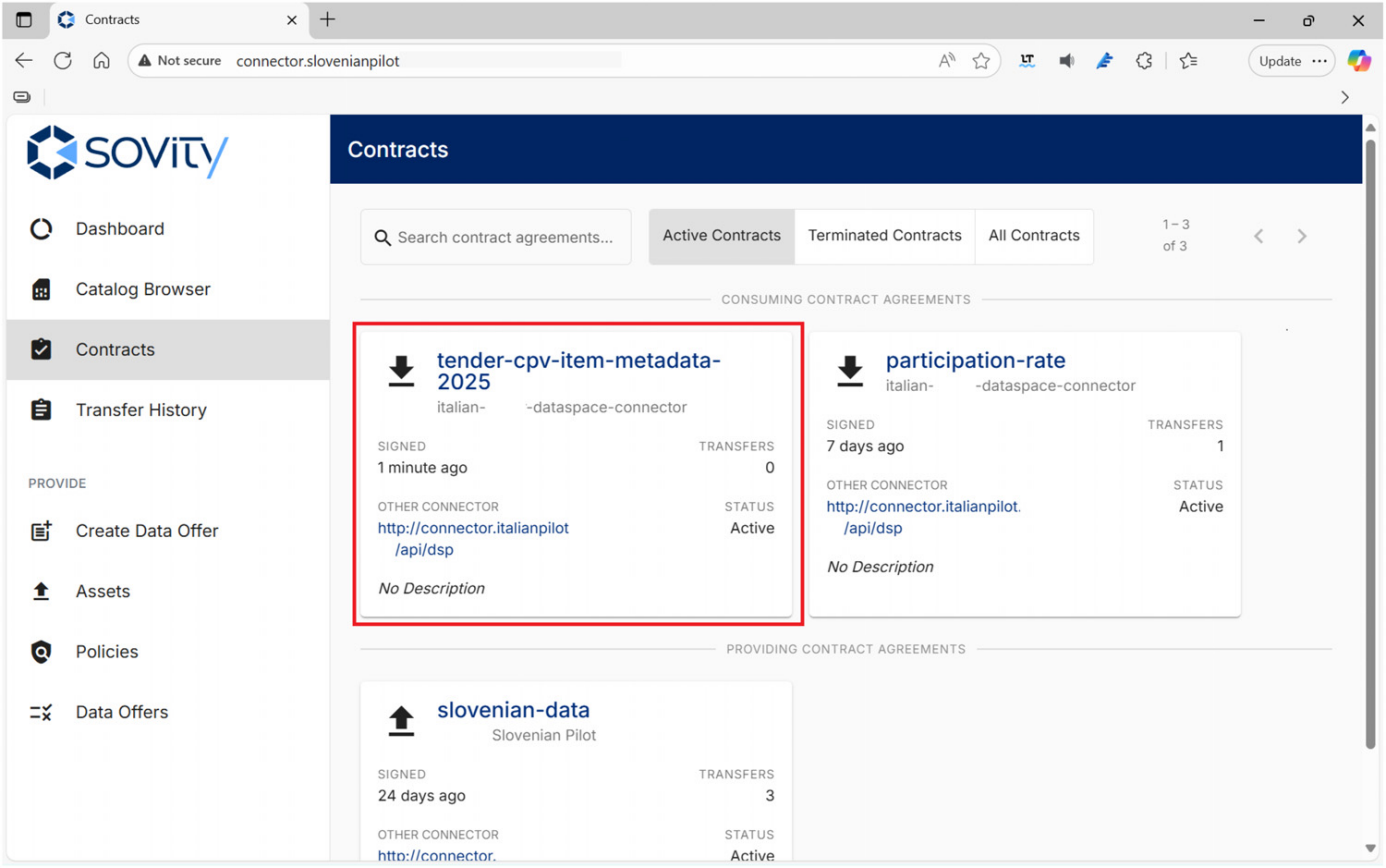
Screenshot from the Slovenian participant’s Contracts view.

**Fig. 9:**
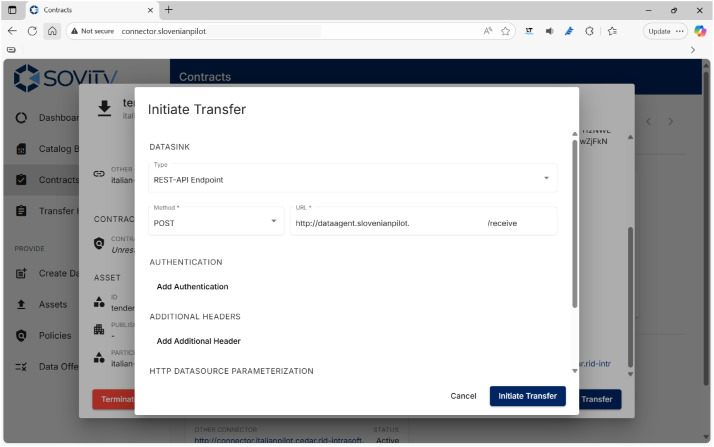
Screenshot from the data transfer process by the Slovenian public procurement entity.

Since the dataset was published without restrictions, the contract negotiation happens automatically. There is no need for manual approval or policy verification. Once the negotiation completes, the contract is established, and the Slovenian end-user gains access to the dataset. The user can now navigate to the “Contracts” tab (Fig. 8), which lists all active agreements. From this section, they can trigger the actual data transfer. To receive the dataset, the end-user provides the local Data Exchange Agent’s receiving endpoint, formatted as http://local-data-exchange-agent/receive (Fig. 9).

**Fig. 10:**
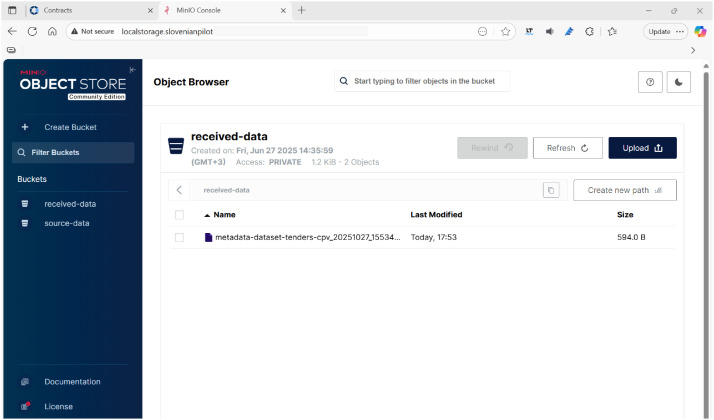
Screenshot from the Slovenian participant’s MinIO ”Received Data” bucket.

After this step, the data transfer is executed successfully. The dataset appears in the Slovenian entity’s MinIO local storage, inside the “received-data” bucket (Fig. 10). The file keeps the same name as the original CSV, with an added timestamp showing the moment it was received. The end-user can now download the file directly from MinIO and utilize it for analysis or integration into their local systems.

**Fig. 11:**
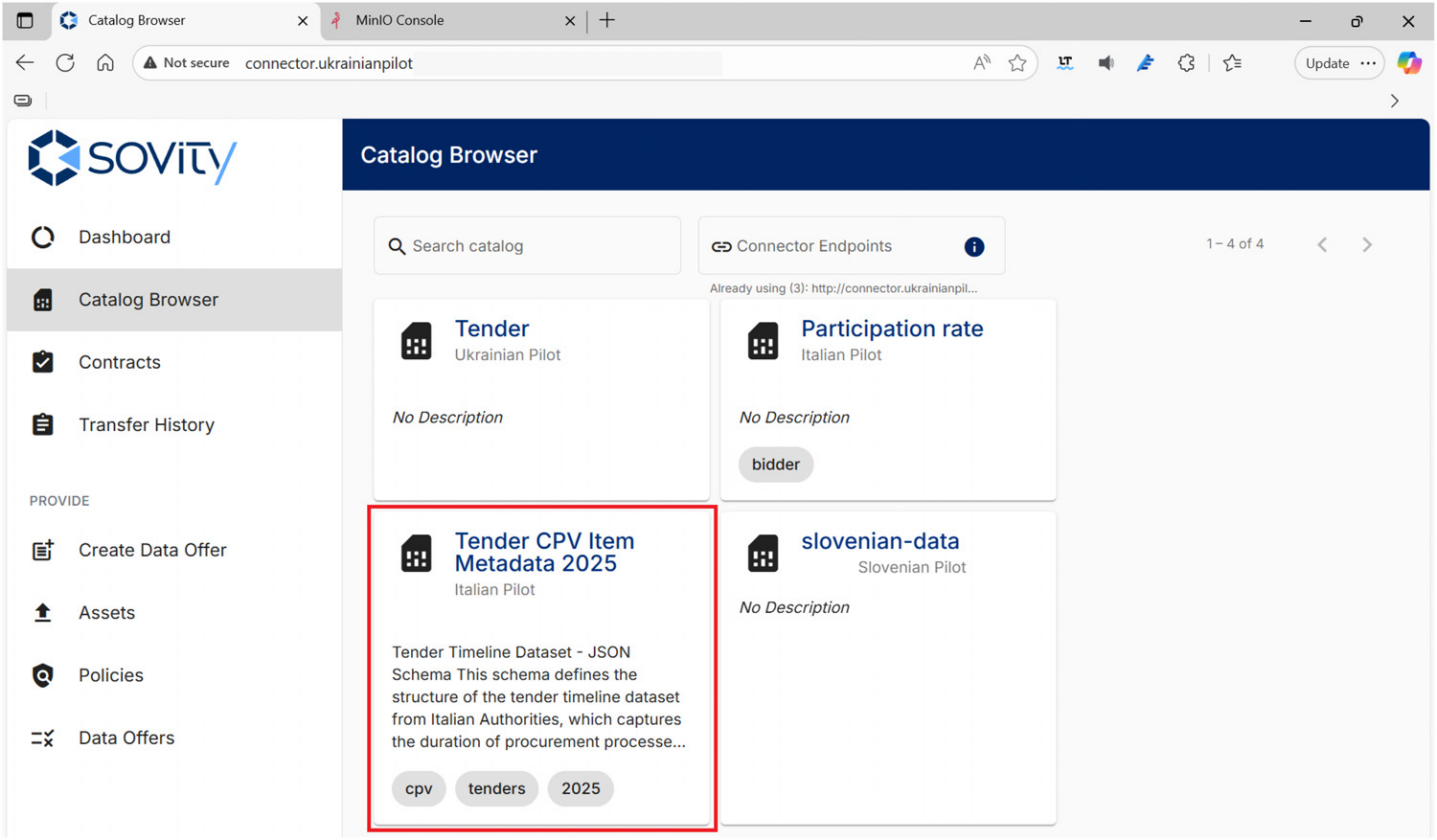
The Ukrainian participant’s Catalog Browser view.

The Ukrainian public procurement entity follows the same process as the Slovenian one. The end-user logs into their local connector UI and opens the “Catalog Browser” to explore available assets (Fig. 11). They find the data offer published by the Italian entity and initiate a contract negotiation. Since the offer is unrestricted, the agreement is completed automatically.

**Fig. 12:**
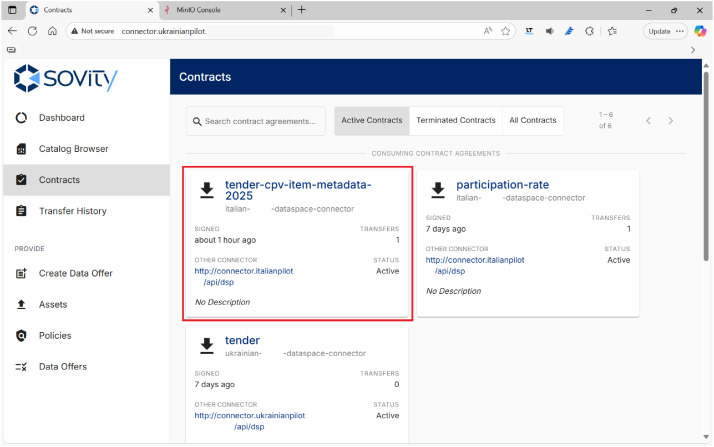
The Ukrainian participant’s Contracts view.

**Fig. 13:**
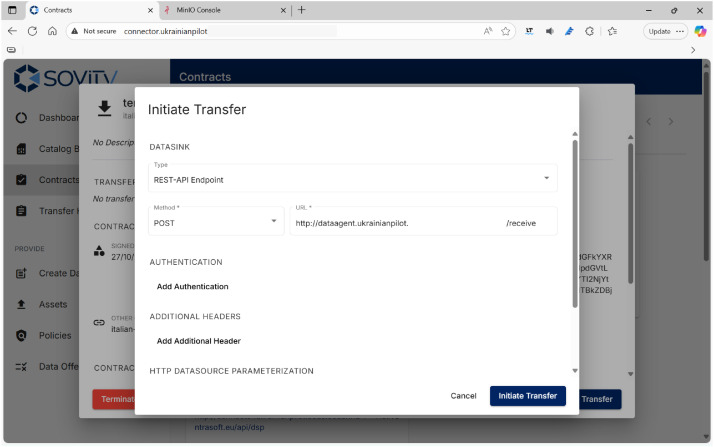
The Ukrainian participant’s data transfer process.

**Fig. 14:**
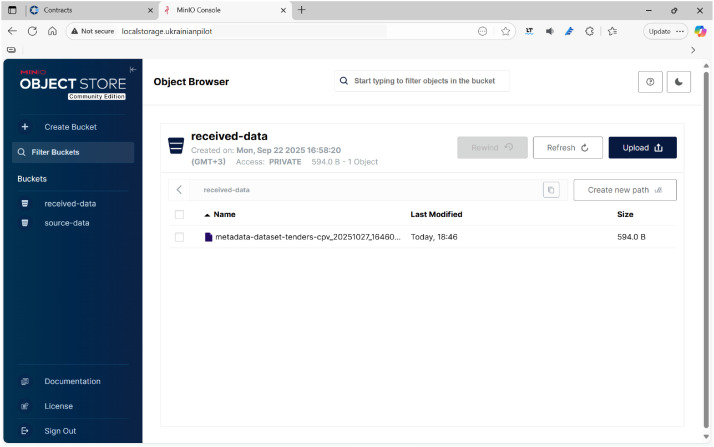
The Ukrainian participant’s MinIO ”Received Data” bucket.

The end-user then goes to the “Contracts” tab (Fig. 12) and uses their local Data Exchange Agent’s receiving endpoint to retrieve the dataset (Fig. 13). Once the transfer is done, the CSV file appears in their MinIO “received-data” bucket, with the same filename as the original plus a timestamp (Fig. 14). The dataset is now ready for local analysis or further use within the Ukrainian entity’s environment.

The prototype deployment showed that a minimal (but well-structured) dataspace setup can work effectively in real public sector environments. The tested MVD showed that secure and (potentially) policy-driven data exchange is achievable without heavy infrastructure or complex integration steps. The DAPS-managed authentication and the connectors’ automated negotiation flow kept data exchanges secure and traceable without creating extra burden for end-users. Even with limited resources, participants could share and retrieve datasets with full control, regardless of their technical capacity. This enhances the assumption that the MVD concept can serve as a practical entry point for public administrations wishing to engage in trusted cross-border data sharing, without needing to overhaul their existing systems.

In practical terms, the MVD proved directly relevant to the context of public procurement, where multiple organizations exchange structured information daily. The ability to share datasets across national boundaries, under controlled conditions, demonstrates a tangible step toward interoperability. Ministries or contracting authorities could use such a setup to share public procurement-related indicators, contract metadata, or supplier / bidder performance data safely and verifiably. The pilot shows that trusted exchange can happen without central intermediaries, relying only on open standards and clear access rules. This makes the MVD a realistic starting point for scaling data sharing across broader public administration networks, while keeping governance simple and transparent.

## 6 Conclusion

The prototype deployment of the Minimum Viable Dataspace confirmed that a simple, standards-based setup can enable trusted and policy-driven data sharing in the public sector. The testing phase showed that each component – DAPS, the sovity-based dataspace connectors, and the Data Exchange Agents – worked together effectively in a distributed environment. Public procurement entities from three countries were able to publish, negotiate, and exchange data securely without external intermediaries. The process remained transparent, traceable, and aligned with European data-sharing principles. These results indicate that the MVD can serve as a realistic aid for current and future Common European Data Spaces, starting from small, controlled deployments that can grow over time.

Despite these promising results, the current implementation faces certain limitations. First, the system has not yet been tested with high data volumes or continuous data streams. This could challenge the capacity of the existing pipeline. Larger datasets may require a more advanced data management layer, or integration with scalable storage frameworks. Another limitation lies in the testing process itself. Since the participating entities are public administrations, real-world testing requires formal approval, which can delay technical validation. Furthermore, an additional limitation is that connector configuration and policy management still demand manual input, making it harder for some users (who are unrelated to such concepts) to deploy or maintain their environments without support. Moreover, the EDC ecosystem is transitioning toward Verifiable Credentials. Since the current MVD’s trust infrastructure relies on DAPS for identity and authorization management, it could affect long-term compatibility.

These issues highlight areas where further development and simplification are needed before wider adoption. In addition, the present prototype targets a small number of participants, along with short-lived pilots. This means that it does not yet address crucial operational concerns. Onboarding and de-onboarding at scale, long-term operation of connectors and agents, automated management of certificate and credential lifecycles, updates, and revocation, are essential aspects for production-grade data spaces. However, they are considered to be beyond the scope of the current implementation, but should be studied as part of this work’s further development.

It should be noted that an MVD has limited value if it remains isolated from a broader scalability path. The architectural choices made in this work, adhering to open standards, using open-source components and aligning with IDSA and CEDS principles, are deliberate. They preserve compatibility and reduce the cost of future transitions. Public sector actors adopting this setup should treat it as a starting point, complemented by a clear roadmap toward full dataspace participation, rather than a self-contained solution.

Future steps will focus on strengthening automation and usability. A Federated Catalog software tool, also proposed as part of a wider dataspace, will be introduced to automatically collect metadata from all connectors and redistribute it across participants, providing synchronized visibility in the catalog browser. A new, user-friendly interface will also be developed, inspired by (but distinct from) the current sovity software’s UI, with an emphasis on clarity and ease of use. Plans are underway for federation with the Public Procurement Data Space initiative, with an aim for the proposed MVD to act as a trusted node within the wider European ecosystem. All future iterations of this MVD should also monitor and align with the EDC ecosystem’s evolution, aiming to ensure continued interoperability within the broader dataspace landscape. Finally, the integration of generative AI tools is being explored. These tools could allow local, offline LLM-based interactions that guide users in publishing, discovering, and managing data assets more intuitively [66].

In conclusion, this work’s proposed MVD represents a practical and achievable step toward realizing the vision of interoperable and trusted European data spaces. It aims to interconnect public procurement entities across the European Union, in order to enable secure and cross-border data exchange. It does so by combining simplicity with compliance, and demonstrating how minimal setups can bring value to public institutions. With continued refinement and expansion, it may evolve into a sustainable foundation for data-driven collaboration across Europe’s public sector.
